# A Review of Traditional Chinese Medicine in Treating Renal Interstitial Fibrosis via Endoplasmic Reticulum Stress-Mediated Apoptosis

**DOI:** 10.1155/2021/6667791

**Published:** 2021-05-15

**Authors:** Yu Liu, Dan-qian Chen, Jing-xue Han, Ting-ting Zhao, Shu-ju Li

**Affiliations:** ^1^Beijing Key Lab for Immune-Mediated Inflammatory Diseases, Institute of Clinical Medical Sciences, China-Japan Friendship Hospital, Beijing 100029, China; ^2^Heilongjiang Academy of Chinese Medical Sciences, Harbin 150001, China

## Abstract

Renal interstitial fibrosis (RIF) is the main pathological manifestation of end-stage renal disease. Recent studies have shown that endoplasmic reticulum (ER) stress is involved in the pathogenesis and development of RIF. Traditional Chinese medicine (TCM), as an effective treatment for kidney diseases, can improve kidney damage by affecting the apoptotic signaling pathway mediated by ER stress. This article reviews the apoptotic pathways mediated by ER stress, including the three major signaling pathways of unfolded protein response, the main functions of the transcription factor C/EBP homologous protein. We also present current research on TCM treatment of RIF, focusing on medicines that regulate ER stress. A new understanding of using TCM to treat kidney disease by regulating ER stress will promote clinical application of Chinese medicine and discovery of new drugs for the treatment of RIF.

## 1. Introduction

Renal interstitial fibrosis (RIF) is a common pathologic process in which various kidney diseases progress to end-stage renal disease (ESRD) [1]. The degree of RIF is related to the severity of renal damage [2, 3]. RIF is characterized by excessive accumulation of extracellular matrix (ECM) in the renal interstitium, which leads to fibroblast hyperplasia, renal interstitial inflammatory cell infiltration, and renal tubular atrophy [4]. The gold standard for assessing RIF is pathologic evaluation after tissue biopsy. Evaluations may include staining with hematoxylin and eosin, Masson trichrome, or sirius red. At the same time, some noninvasive assessments, such as magnetic resonance imaging and ultrasound, are used to determine the extent of RIF through examining the shape of the kidney, renal stiffness, and renal blood flow [5]. Current treatment for RIF is multifactorial, including the use of renin-angiotensin system inhibitors and lipid therapy for blood pressure control, and dietary restrictions. Although these strategies have protective effects on the kidneys, the incidence of ESRD is still high [6].

The pathogenesis of RIF is complicated. Previous studies have made great progress in understanding effector cells, extracellular matrix, and cytokine involvement [7, 8]. A large number of studies have demonstrated that multiple signal transduction pathways are involved in the process of renal fibrosis. At present, the more plausible pathways include bone morphogenic protein (BMP) [9], platelet-derived growth factor (PDGF) [10], hepatocyte growth factor (HGF) [11], vascular endothelial growth factor (VEGF) [12], TGF-*β*/Smad [13, 14], Wnt [15, 16], and Hedgehog [17, 18]. However, the molecular mechanisms of RIF need to be further elucidated. Extensive research on stress responses at the cellular level has found organelle stress is associated with many kidney diseases [19]. An area of intense study is the apoptotic pathway induced by endoplasmic reticulum stress (ER stress) and its relationship with RIF [19, 20]. It has been reported that the TGF-*β*1/Smad2/3 signaling pathway interacts with ER stress and can regulate the expression of podocyte *α*-smooth muscle actin and synergistically induce podocytes to undergo epithelial-mesenchymal transition [21]. By interfering with the occurrence of ER stress or inhibiting the activity of key enzymes in the apoptosis signaling pathway mediated by ER stress, RIF can be attenuated, suggesting that ER stress is a new fibrosis factor in the complex network of renal fibrosis [22, 23].

In China, traditional Chinese medicines (TCMs) have a long history of treating kidney disease and are still used as an alternative therapy for renal disorders. TCM has unique advantages in improving the quality of life and long-term survival of patients [24]. Clinical trials have validated that a large number of Chinese herbal formulas are effective for RIF, with active ingredients, such as tripterygium glycosides [25], resveratrol [26, 27], and astragaloside [28]. TCMs can inhibit ER stress by abating the apoptosis pathway, which may explain how herbal medicines exert their renal protective function in the treatment of RIF [22, 29, 30]. This article reviews recent research on TCM in the treatment of RIF, focusing on the core mechanism of ER stress-mediated apoptosis.

## 2. ER Stress, Apoptosis, and RIF

### 2.1. Endoplasmic Reticulum and ER Stress

The endoplasmic reticulum (ER) is the largest organelle in the cell cytoplasm. It coordinates at least one-third of protein synthesis, folding, and transporting in the cell, and is important for regulating cell homeostasis [31]. The ER has a strong homeostasis system, and the stability of its internal environment is fundamental for ER function. When abnormal proteins, such as misfolded proteins and unfolded proteins, in the ER cavity are increased or calcium ion balance is changed, ER stress can be induced. Various endogenous and exogenous physiologic and pathologic factors can cause ER stress, such as hypoxia, viral infection, nutritional deficiencies, chemical drugs, free radical attack, calcium ion imbalance, and inflammatory challenges [32]. Three kinds of ER stress are recognized: sterol regulatory element and protein-mediated regulatory response, unfolded protein reaction (UPR), and endoplasmic reticulum overload response [33]. ER stress usually refers to UPR that occurs when misfolded or unfolded proteins in the ER increase and activate the stress signal, which is then transmitted to the nucleus through the ER membrane.

The main function of UPR is to restore ER homeostasis and reduce stimulation of the ER caused by internal and external environmental changes. It is the adaptive response of cells to external stimuli, including reducing protein synthesis, promoting the degradation of abnormal proteins, and participating in the correct folding of proteins [32]. UPR mediates three signaling pathways, which are mainly decomposed by three factors: protein kinase RNA-like endoplasmic reticulum kinase (PERK), activated transcription factor 6 (ATF6), and inositol-requiring enzyme 1 (IRE1) [34]. When the ER is in a steady state, all three sensor proteins are in an inactive state by binding to molecular chaperone glucose regulatory protein 78/immunoglobulin heavy-chain-binding protein (GRP78/Bip). When ER homeostasis imbalance induces ER stress, the three receptor proteins separate from GRP78/Bip, leading to activation of receptor proteins. After their activation, PERK, IRE1, and ATF6 activate three signaling pathways: PERK/eIF2*α*/ATF4, IRE1/XBP1, and ATF6. GRP78/Bip is the hallmark protein of the ER stress response. Furthermore, ER stress cannot simultaneously activate three UPR signaling branches. Activation of ATF6 and IRE1 occurs immediately and decays over time through an undefined mechanism in ER stress, while PERK is continuously activated [35, 36]. When the high intensity of ER stress occurs or persists, ER-related molecules cannot restore homeostasis and induce cellular apoptosis [37].

### 2.2. ER Stress-Mediated Apoptosis Pathway

The ER stress-mediated apoptosis signaling pathway is mainly activated through CHOP, JNK, and caspase signaling pathways [38, 39]. The main concern is the CHOP signaling pathway, which can also trigger the caspase signaling pathway. Through the binding site within the CHOP promoter, the UPR signaling pathway leads to the initiation of CHOP transcription when under chronic or overwhelming ER stress [40]. CHOP is also known as growth arrest and DNA damage-inducible gene 153 (*GADD153*). It is a member of the C/EBP family and is a special transcription factor that exists in the ER. CHOP can trigger the endogenous apoptosis pathway and promote cell apoptosis by inhibiting the upregulation of BCL-2 [41]. CHOP can also upregulate the expression of TRB3, thus preventing Akt phosphorylation [42–44]. CHOP can also initiate the exogenous apoptosis pathway through DR4 and DR5 [45] and can trigger the ERO1*α*-IP3R-Ca^2+^-CaMKII pathway, leading to the accumulation of reactive oxygen species (ROS), which are involved in cell apoptosis [46]. The function and specificity of CHOP are important for the execution of the ER stress-induced apoptosis signaling pathway.

**Figure 1:**
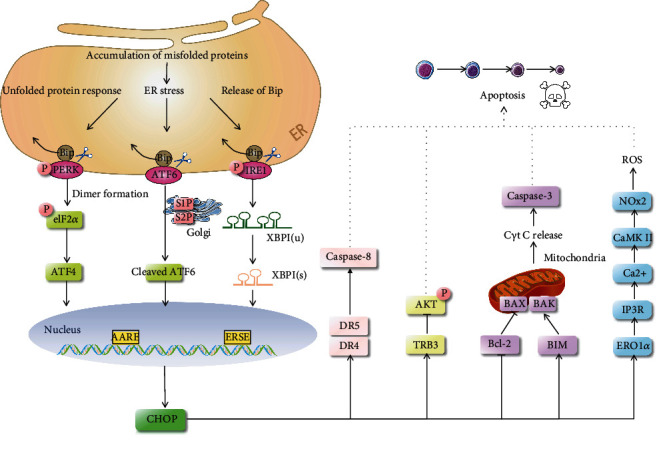
Mechanisms of ER stress-mediated apoptosis. Once PERK is activated, eIF2*α* is phosphorylated to achieve ATF4 translation and CHOP activation. Activation of the IRE1*α* domain processes uncut XBP1mRNA to produce activated XBP1(s), which enters the nucleus and controls the expression of CHOP. ATF6*α* is transported to the Golgi apparatus, where it is processed by the proteases SP1 and SP2 to produce cytoplasmic fragment ATF6, which regulates CHOP activation in the nucleus. CHOP can trigger the endogenous apoptosis pathway and promote cell apoptosis by inhibiting the upregulation of BCL-2. CHOP can also upregulate the expression of TRB3, preventing Akt phosphorylation. CHOP can also initiate the exogenous apoptosis pathway through DR4 and DR5 and can additionally trigger the ERO1*α*-IP3R-Ca^2+^-CaMKII pathway.

Three signaling pathways mediated by UPR can induce CHOP transcription. The PERK pathway is dominant in CHOP activation. In ER stress, PERK is activated and phosphorylates eukaryotic translation initiation factor 2*α* (eIF2*α*), which weakens overall protein synthesis. ATF4 is then transferred to the nucleus after eIF2*α* activation and transcriptionally upregulates CHOP and many UPR genes that are essential for amino acid metabolism and redox processes [40]. When cells are under stress, PERK upregulates the expression of CHOP to promote apoptosis; the CHOP pathway is the main pathway by which ER stress induces apoptosis [27, 39]. The activation of IRE1 is similar to that of PERK. Its luminal domain is first dimerized and then transautophosphorylated. Activated IRE1 uses cytoplasmic RNase domain to cut a 26-nucleotide intron from unspliced X-box-binding protein 1 (XBP1) mRNA to produce spliced XBP1(s). IRE1 also mediates the IRE1-dependent decay of selective mRNAs. XBP1(s) enters the nucleus and induces transcription of genes related to protein folding ability and degradation of the ER. Thus, XBP1(s) upregulates CHOP expression [47]. In the ATF6 branch, type II ER localization protein ATF6 is transported to the Golgi apparatus, where it is used by Site-1 and Site-2 proteases (SP1 and SP2). In this process, a cytoplasmic fragment of ATF6f is produced and enters the nucleus to regulate expressions of target genes including *Bip* and *CHOP* [48] (Figure 1).

### 2.3. ER Stress-Mediated Apoptosis in RIF

Investigations have confirmed that ER stress is a key factor in renal tubular epithelial cell atrophy and RIF [49, 50]. The unilateral ureteral obstruction (UUO) model found that the expression of ER stress-related proteins, such as GRP78, CHOP, and caspase-3, is significantly increased [51]. Furthermore, knockout of the *CHOP* gene not only reduces apoptosis and oxidative stress in experimental renal fibrosis but also reduces local inflammation and attenuates UUO-induced renal fibrosis, indicating that UUO-induced tubular epithelial cell apoptosis is related to the activation of the apoptosis pathway of ER stress [27]. Chronic interstitial nephropathy caused by some drugs is also related to apoptosis of renal tubular epithelial cells. Studies have shown that cyclosporine A can upregulate the expression of apoptosis proteins, such as GRP78, CHOP, and caspase-3, and promote apoptosis of renal tubular epithelial cells through the ER stress pathway [52–54]. This apoptosis is also time- and dose-dependent, and the degree of apoptosis is correlated with the stage of kidney disease [55]. Therefore, the ER stress-mediated apoptosis signaling pathway is one of the important mechanisms of RIF.

## 3. Mechanisms of TCMs in Treating RIF by Affecting the ER Stress-Mediated Apoptosis Pathway

RIF is the result of multiple mechanisms. TCMs have unique advantages in the prevention and treatment of RIF. Multiple compounds in TCMs can intervene in the occurrence and progression of RIF by acting on multiple targets. Traditional herbal formulas have compounds that are rich in antioxidants and minerals, which attenuate oxidative stress/nitrosative stress/ER stress by improving protein folding and cell survival. One such formula is Shenkang injection, which contains the extracts of four medicinal plants: rhubarb root and rhizome (*Rheum palmatum* L.), astragalus root (*Astragalus membranaceus* (Fisch.) Bunge), salvia root (*Salviae miltiorrhizae* Bunge), and safflower (*Carthamus tinctorius* L.) [56]. Studies have found that certain Chinese medicines appear to be effective for RIF by intervening in the apoptosis signaling pathway mediated by ER stress. Single compounds extracted from herbs, whole herbs, and herbal formulas have been investigated (Table 1). In the following, we summarize these investigations.

### 3.1. Pure Compounds

Resveratrol (RSV) is a natural polyphenol that exists in a variety of edible plants, such as knotweed, grapes, peanuts, and berries. RSV is reported to effectively inhibit obstructive RIF [76]. In the UUO model, RSV can block the expression of eIF2*α* and ATF4 proteins, thereby preventing excessive apoptosis of renal tubular epithelial cells and delaying the development of RIF [26]. In diabetic kidney disease (DKD) rats induced by streptozotocin (STZ), RSV treatment has been found to be renal protective by reducing urinary protein level and kidney damage in addition to attenuating ER stress through the PERK pathway [57].

Stachytine is the main biologically active ingredient extracted from the TCM Chinese motherwort (*Leonurus sibiricus* L.) and has been found to have antifibrotic and anti-inflammatory effects [77]. In the UUO model, stachytine not only inhibits the expression and activation of caspase-12 but also reduces the expression of PERK, CHOP, and caspase-3 and interferes with the ER stress-mediated apoptosis pathway, eventually inhibiting apoptosis of renal tubular epithelial cells and delaying the occurrence of RIF [58, 59].

Emodin, a phenolic compound extracted from rhubarb, knotweed, and other herbs, is widely used to reduce inflammation and inhibit cell proliferation and ER stress [78, 79]. In KKAy model mice, emodin increased the expression of podocyte nephrin, significantly reduced apoptosis and ER stress markers (GRP78), and also reduced p-PERK, p-eIF2*α*, ATF4, and CHOP expression. Results of in vitro experiments found that emodin treatment reduced both the level of GRP78 and apoptosis of podocytes cultured with high glucose by inhibiting the upregulation of phosphorylated PERK, P-eIF2*α*, ATF4, and CHOP. These findings are consistent with the results of animal experiments in which emodin was shown to reduce podocyte apoptosis induced by ER stress through inhibition of the PERK-eIF2*α* pathway, downregulation of the expression of CHOP, thus ultimately delaying progression of renal fibrosis [63].

Ginsenoside-Rg1 (G-Rg1) is the main active component in ginseng (*Panax ginseng* C. A. Mey). G-Rg1 has antioxidative, antiproliferation, and antiapoptosis effects and can significantly inhibit organ fibrosis [80]. G-Rg1 has been shown to inhibit excessive apoptosis of renal tubular epithelial cells and delay progression of renal fibrosis by reducing expressions of GRP78 and its downstream proapoptotic factors CHOP and caspase-12 [51]. In addition, G-Rg1 treatment in chronic CsA nephropathy models can reduce expressions of GRP78 and CHOP and can inhibit apoptosis of renal tubular cells triggered by ER stress [60].

Astragaloside IV (AS-IV) is extracted from the Chinese herbal medicine astragalus (*Astragalus membranaceus* (Fisch.) Bunge), which is widely used to treat diabetic nephropathy and other kidney diseases in China. AS-IV treatment can prevent STZ-induced rat mesangial matrix expansion. It can significantly inhibit phosphorylation of eIF2*α*, PERK, and JNK both in vivo and in vitro and significantly inhibit expressions of GRP78 and ORP150 and prevent podocyte apoptosis induced by tunicamycin in vitro, while reducing the expression of CHOP and caspase-3 [29]. Another study came to the same conclusion that in the STZ-induced rat, AS-IV has a protective effect on ER stress-induced podocyte apoptosis by inhibiting the PERK-ATF4-CHOP pathway [61]. Furthermore, AS-IV treatment can inhibit apoptosis of renal tubular epithelial cells induced by ER stress by downregulating expressions of p-PERK, ATF4, and CHOP [62].

Curcumin is a natural polyphenol compound present in the rhizome of turmeric (*Curcuma longa* L.). Curcumin has anti-inflammatory, antioxidant, and antiapoptotic properties and has excellent safety [81]. Curcumin has been shown to effectively prevent and treat renal fibrosis by attenuating the increased protein expression of mitogen-activated protein kinases (MAPKs), such asp-JNK, p-ERK1/2, GRP78, and CHOP in the kidney tissue of nonalcoholic steatohepatitis (NASH) mice [82]. In addition, curcumin also reduced NASH renal cell apoptosis signaling protein (cleaved caspase-3, cleaved caspase-12). These results indicate that curcumin protects against the development of chronic kidney disease in NASH mice by reducing ER stress-induced apoptosis and MAPK signal transduction [30].

Natural flavonoid morin hydrate (MH) is a compound isolated from white mulberry (*Morus alba* L.) and other fruits such as apple (*Malus*), Osage orange (*Maclura pomifera*), guava (*Psidium guajava*), and fig (*Ficus carica*). MH has a wide range of pharmacologic activities, including antioxidant, anti-inflammatory, antiapoptotic, and antiautophagy [83–85]. In cisplatin-treated HEK-293 cells and mouse kidneys, ER stress was induced through increasing intracellular ROS and decreasing antioxidant enzymes. After exposure to MH, ER stress markers, such as the expression of PERK, IRE-1*α*, p-eIF2*α*, CHOP, and casp-12, were all attenuated. Furthermore, MH may inhibit the mechanism of autophagy and cell death mediated by ER stress, indicating that MH protects against cisplatin-treated kidney damage [64].

Withaferin A (WA) is a compound in ashwagandha (*Withania somnifera*) that has antioxidant and anticancer activities [86]. Ashwagandha is commonly used in India as a traditional medicine and dietary supplement [87]. Studies have demonstrated that WA improves symptoms of several chronic diseases and cancers [88, 89]. In laboratory mice, WA significantly reduced histopathologic changes and collagen deposition of UUO in the kidneys and reduced UUO mouse endoplasmic reticulum stress-related molecules (GRP78, GRP94, ATF4, CHOP, phosphorylated eIF2*α*, and cleaved caspase-12). Thus, WA is able to protect against the progression of chronic kidney disease by improving endoplasmic reticulum stress-related apoptosis, inflammation, and fibrosis [65].

### 3.2. Extracts

*Cordyceps sobolifera* (CS) is a TCM that has long been used to treat kidney diseases. Water extract of fermented decoction of CS can attenuate lipopolysaccharide-induced ER stress and tissue damage and reduce cell apoptosis through reducing GRP78, caspase-12, Bax/Bcl-2 ratio, and caspase-3 expression [66].

Ginkgo biloba extract (GBE) is derived from ginkgo leaf (*Ginkgo biloba* L.). Its main active ingredients are flavonoids and terpenoid lactones. GBE has a wide range of clinical applications and is mostly used in the treatment of cardiovascular and cerebrovascular diseases [90]. In DKD mice induced by STZ and high-fat diet, the expression of ER stress markers GRP78 and ATF6 in renal tissue decreased after GBE treatment. Moreover, GBE was found to reduce urine *β*2-MG, RBP4, and NGAL levels in mice, indicating that GBE attenuates renal tubular damage in DKD mice [67].

Lemongrass (*Cymbopogon citratus* (DC.) Stapf) is commonly used in traditional medicine for the management of a number of diseases including diabetes mellitus [91, 92]. Methanolic extract of lemongrass leaves was found to reduce ER stress induced by streptozotocin in rats by downregulating GRP78 and upregulating Nrf2 signaling. This may be the effects of the flavonoids apigenin, quercetin, and kaempferol that are found in lemongrass [68].

### 3.3. Chinese Herbal Formulas

Several Chinese herbal formulas are known to treat RIF through inhibiting ER stress. Chaiqin Chengqi Decoction is comprised of the following TCMs: bupleurum root (*Bupleurum chinense* DC.), scutellaria root (*Scutellaria baicalensis* Georgi), rhubarb root and rhizome (*Rheum palmatum* L.), crystallized sodium sulfate, magnolia bark (*Magnolia officinalis* Rehder & E.H.Wilson), bitter orange (*Citrus aurantium* L.), virgate wormwood (*Artemisia scoparia* Waldst. & Kitam.), and gardenia fruit (*Gardenia jasminoides* J.Ellis). In an animal model of acute kidney injury caused by acute pancreatitis, Chaiqin Chengqi Decoction was found to inhibit renal ER stress indicators (BIP, XBP1s, and CHOP) and apoptotic protein caspases (caspase-9 and Cle-caspase-3) and reduce pathophysiologic changes and cell apoptosis in the kidney, thereby restoring kidney function. In vitro experiments found that both TNF-*α* and IL-6 activate ER stress of HK-2 cells. Chaiqin Chengqi Decoction can restore cellular ER stress indicators and the expression level of caspases and reduce the number of dead HK-2 cells [69].

Shenshuaiyin Decoction consists of the following TCMs: astragalus root (*Astragalus membranaceus* (Fisch.) Bunge), prince's feather (*Polygonum orientale* L.), pseudostellaria root (*Pseudostellaria heterophylla* (Miq.) Pax), self-heal spike (*Prunella vulgaris* L.), round cardamom (*Amomum kwangsiense* D. Fang & X.X.Chen), white atractylodes rhizome (*Atractylodes macrocephala* Koidz.), cuscuta seed (*Cuscuta chinensis* Lam.), smooth greenbrier rhizome (*Smilax glabra* Roxb.), cogon grass root (*Imperata koenigii* (Retz.) P. Beauv.), agastache (*Agastache rugosa* (Fisch. & C.A.Mey.) Kuntze), rhubarb (*Rheum palmatum* L.), pinellia tuber (*Pinellia ternata* (Thunb.) Breit.), salvia root (*Salvia miltiorrhiza* Bunge), leech (*Hirudo nipponica* Whitman), curcuma rhizome (*Curcuma zedoaria* (Christm.) Roscoe), and Chinese soft-shelled turtle shell (*Trionyx sinensis* Wiegmann). Shenshuaiyin Decoction has been found to reduce kidney damage and improve kidney function. In the adenine-induced renal failure rat model, treatment with Shenshuaiyin Decoction attenuated expression levels of ATF6, CHOP, and caspase-3 protein in kidney tissues, indicating that this medicine may protect kidney function by affecting the ATF6/CHOP pathway and reducing renal cell apoptosis [70].

Tongluo Baoshen Formula is comprised of the following TCMs: astragalus root (*Astragalus membranaceus* (Fisch.) Bunge), cornus fruit (*Cornus officinalis* Sieb. et Zucc.), privet fruit (*Ligustrum lucidum* W.T.Aiton), salvia root (*Salviae miltiorrhizae* Bunge), chuanxiong root (*Ligusticum chuanxiong* Hort.), leech (*Hirudo nipponica* Whitman), cogongrass (*Imperata cylindrica* (L.) P. Beauv.), plantago seed (*Plantago asiatica* L.), and rhubarb (*Rheum palmatum* L.). Research has shown that Tongluo Baoshen Formula can significantly reduce the expression of GRP78 and p-JNK protein in the kidney tissue of diabetic rats, inhibit ER stress response and its induced JNK apoptosis signaling pathway, reduce renal cell apoptosis, and protect kidney function [71].

Danggui Buxue Decoction is a simple formula that has only two TCMs: astragalus root (*Astragalus membranaceus* (Fisch.) Bunge) and tangkuei (*Angelica sinensis* (Oliv.) Diels). In the STZ-induced diabetic rat model, Danggui Buxue Decoction treatment reduced expressions of p-IRE1*α* and p-JNK protein as well as apoptosis in kidney tissue, thus lessening the ER stress response of the kidney under high glucose condition and protecting the kidney [72].

Manshen Kangning Formula is yet another Chinese herbal prescription that treats RIF. The formula consists of the following TCMs: astragalus root (*Astragalus membranaceus* (Fisch.) Bunge), codonopsis root (*Codonopsis pilosula* (Franch.) Nannf.), poria (*Poria cocos* (Schw.) Wolf), epimedium aerial parts (*Epidemium*), cornus fruit (*Cornus officinalis* Sieb. et Zucc.), ophiopogon tuber (*Ophiopogon japonicas* (Thunb.) Ker-Gawl.), rehmannia root (*Rehmannia glutinosa* Libosch), salvia root (*Salviae miltiorrhizae* Bunge), cuttlefish bone (*Sepiella maindroni* Rochebruneor Sepia esculenta Hoyle), and rhubarb root and rhizome (*Rheum palmatum* L.). In the adenine-induced renal failure rat model, Manshen Kangning Formula reduced apoptosis in kidney tissue and expressions of GRP78, GRP78mRNA, ATF-4mRNA, and CHOPmRNA. Therefore, the formula has an inhibitory effect on the overexpression of the PERK-eIF2*α*-ATF4-CHOP pathway, thus attenuating the ER stress response and protecting renal function [73].

Finally, another Chinese formula that has been studied for its ability to treat nephropathy is Huaiqihuang. The formula consists of the fungus trametes, goji berry (*Lycium barbarum* L.), and polygonatum rhizome (*Polygonatum sibiricum* F.Delaroche), which have long been used in China to treat kidney disease. Research has shown that mechanisms by which they exert their effects include regulating oxidative stress and inhibiting apoptosis [93]. Other studies have shown that the formula protects podocytes by suppressing the p-ERK/CHOP signaling pathway and reversing mercury-induced upregulation of GRP78 and reducing dysfunction of podocyte apoptosis and DNA damage [74, 75]. Thus, Huaiqihuang inhibits cell apoptosis by inhibiting ER stress and has a therapeutic effect on podocyte dysfunction-related renal diseases.

## 4. Discussion and Conclusion

Traditional Chinese medicines (TCMs) have been used effectively in China for centuries to treat and delay progression of kidney diseases. The therapeutic value of TCMs and their isolated compounds in the treatment of RIF has been demonstrated by a large number of animal experiments based on modern pharmacologic research. However, clinical application and research on TCMs aimed at regulating ER stress are limited; therefore, well-designed and executed clinical studies are needed. Although the TCMs described above are not toxic at normal therapeutic doses, we must be aware that some TCMs may cause significant toxicity, including nephrotoxicity. Biologically active compounds extracted from those TCMs may actually promote excessive ER stress, which may be the important mechanism of their toxicity. To date, most studies on TCMs have shown their protective effect against ER stress in the kidneys. However, further exploration is needed to clarify how TCMs reduce ER stress in renal interstitial fibrosis and thus cell apoptosis.

In this review, we mainly discussed the potential mechanisms of TCMs in the treatment of RIF by regulating cell apoptosis caused by ER stress. Chinese herbal formulas such as Chaiqin Chengqi Decoction, Shenshuaiyin Decoction, Tongluo Baoshen Formula, and various active compounds extracted from TCMs, such as resveratrol, astragaloside IV, and emodin are effective for the prevention and treatment of RIF. However, in-depth investigations are needed to determine the mechanisms of Chinese medicines in regulating ER stress, which may lead to new treatment strategies for RIF.

## Figures and Tables

**Table 1:** Traditional Chinese medicines effective for renal interstitial fibrosis.

	Name	Animal model	Targeted pathway	Targeted protein	Reference
Compounds	Resveratrol	UUO rats		eIF2*α*, ATF4	[26]
		STZ-induced rats	PERK	GRP78, p-PERK, ATF4, CHOP	[57]
	Stachydrine	UUO rats		PERK, CHOP, caspase-3	[58]
		UUO rats		Caspase-12, caspase-9	[59]
	Ginsenoside-Rg1	UUO rats		GRP78, CHOP, caspase-12	[51]
		CsA-induced rats		GRP78, CHOP	[60]
	Astragaloside IV	STZ-induced rats; HG-induced podocytes	PERK-ATF4-CHOP	GRP78, CHOP, ATF4, TRB3	[61]
		STZ-induced rats; TM-induced podocytes		eIF2*α*, PERK, JNK, GRP78, ORP150, CHOP, cleaved caspase-3	[29]
		STZ+HFD-induced rats		GRP78, p-PERK, PERK, ATF4, CHOP, cleaved caspase-3, Bax/Bcl-2 ratio	[62]
	Curcumin	STZ+HFD-induced mice	ER stress-induced apoptosis; MAPK	GRP78, p-JNK, p-PERK1/2, CYP2E1, cleaved caspase-12, cleaved caspase-3, CHOP	[30]
	Emodin	KKAy mice, HG or TM-induced podocytes	PERK	GRP78, p-PERK, p-eIF2*α*, ATF4, CHOP	[63]
	Morin hydrate	CP-induced HEK-293 cells, CP-induced ICR mice		PERK, IRE-1*α*, p-eIF2*α*, CHOP, caspase-12	[64]
	Withaferin A	UUO mouse		GRP78, GRP94, ATF4, CHOP, p-eIF2*α*, and cleaved caspase-12	[65]

Extracts	*Cordyceps sobolifera*	LPS-induced PK1 cells and MDCK cells, LPS-induced rats		GRP78, caspase-12, Beclin-1, caspase-3, PARP, Bax/Bcl-2	[66]
	Gingko biloba	STZ+HFD-induced mice		GRP78, ATF6	[67]
	*Cymbopogon citratus* (DC. Stapf)	STZ-induced rats	NRF2 signaling	GRP78, PERK, IRE-1, p-eIF2*α*, CHOP, ATF4	[68]

Chinese herbal formula	Chaiqin Chengqi Decoction	Taurocholate-induced rats; TNF-*α* or IL-6-treated HK-2		Caspase-9, cleaved caspase-3, GRP78/BIP, IRE1*α*, XBP1, CHOP	[69]
	Shenshuaiyin Decoction	Adenine-induced rats	ATF6/CHOP	ATF6, CHOP, caspase-3	[70]
	Tongluo Baoshen Formula	STZ-induced rats	JNK	GRP78, p-JNK	[71]
	Danggui Buxue Decoction	STZ-induced rats	IRE1*α*-JNK	p-IRE1*α*, p-JNK	[72]
	Manshen Kangning Formula	Adenine-induced rats	PERK-eIF2*α*-ATF4-CHOP	GRP78, CHOP, ATF4	[73]
	Huaiqihuang	MPC5 podocyte	p-ERK/CHOP pathway	p-PERK, PERK, CHOP	[74, 75]

Abbreviations: ATF4: activated transcription factor 4; ATF6: activated transcription factor 6; BCL2: B-cell lymphoma 2; CHOP: transcription factor C/EBP homologous protein; CP: cisplatin; CsA: cyclosporine A; CYP2E1: cytochrome P450 protein; eIF2*α*: eukaryotic translation initiation factor 2*α*; ER: endoplasmic reticulum; GRP78: glucose regulatory protein 78; HEK-293: human embryonic kidney epithelial cell; HFD: high-fat diet; HG: high glucose; HK-2: human renal proximal tubular cell; IRE1*α*: inositol requirement protein 1*α*; JNK: c-Jun N-terminal kinase; LPS: lipopolysaccharide; MAPK: mitogen-activated protein kinase; MDCK: Madin-Darby canine kidney epithelial cell; ORP150: oxygen regulator 150; p-eIF2*α*: phosphorylation-eIF2*α*; p-IRE1*α*: phosphorylation-IRE1*α*; p-JNK: phosphorylation-JNK; PERK: protein kinase RNA-like endoplasmic reticulum kinase; p-PERK: phosphorylated PERK; PK1: porcine kidney epithelial cell; STZ: streptozotocin; TM: tunicamycin; TRB3: tribbles-related protein 3; UUO: unilateral ureteral obstruction; XBP1: X-box-binding protein 1.
